# Settlement-Size Scaling among Prehistoric Hunter-Gatherer Settlement Systems in the New World

**DOI:** 10.1371/journal.pone.0140127

**Published:** 2015-11-04

**Authors:** W. Randall Haas, Cynthia J. Klink, Greg J. Maggard, Mark S. Aldenderfer

**Affiliations:** 1 Department of Anthropology, University of Maryland, 1111 Woods Hall, College Park, MD, 20742, United States of America; 2 Department of Anthropology, SUNY College at Oneonta, 10 Denison Hall, Oneonta, NY, 13820, United States of America; 3 Department of Anthropology, University of Kentucky, 1020-A Export St., Lexington, KY, 40506–9854, United States of America; 4 School of Social Sciences, Humanities, and Arts, University of California Merced, 5200 Lake Road, Merced, CA, 95343, United States of America; University of Gävle, SWEDEN

## Abstract

Settlement size predicts extreme variation in the rates and magnitudes of many social and ecological processes in human societies. Yet, the factors that drive human settlement-size variation remain poorly understood. Size variation among economically integrated settlements tends to be heavy tailed such that the smallest settlements are extremely common and the largest settlements extremely large and rare. The upper tail of this size distribution is often formalized mathematically as a power-law function. Explanations for this scaling structure in human settlement systems tend to emphasize complex socioeconomic processes including agriculture, manufacturing, and warfare—behaviors that tend to differentially nucleate and disperse populations hierarchically among settlements. But, the degree to which heavy-tailed settlement-size variation requires such complex behaviors remains unclear. By examining the settlement patterns of eight prehistoric New World hunter-gatherer settlement systems spanning three distinct environmental contexts, this analysis explores the degree to which heavy-tailed settlement-size scaling depends on the aforementioned socioeconomic complexities. Surprisingly, the analysis finds that power-law models offer plausible and parsimonious statistical descriptions of prehistoric hunter-gatherer settlement-size variation. This finding reveals that incipient forms of hierarchical settlement structure may have preceded socioeconomic complexity in human societies and points to a need for additional research to explicate how mobile foragers came to exhibit settlement patterns that are more commonly associated with hierarchical organization. We propose that hunter-gatherer mobility with preferential attachment to previously occupied locations may account for the observed structure in site-size variation.

## Introduction

Extreme settlement-size variation predicts extreme variation in the rates and magnitudes of many social and ecological processes in human societies including the rates and magnitudes of technological innovation, disease transmission, crime, and wealth [1–8]. Understanding how extreme settlement-size variation self-organizes (sensu [9]) and persists in human societies is therefore relevant to modeling such processes. Yet, the behavioral basis for settlement-size variation remains poorly understood [10–12]. Previous demographic research on hierarchically organized societies has observed that settlement-size variation, whether measured by census counts or areal extents, is heavy-tailed with the largest settlements in the upper tail of the distribution tending to exhibit scale-free, or power-law structure such that f(x) ∝ x^‒α^, where *x* is settlement size and *α* is a scaling exponent [13–15]. Scholars commonly link this variation to central-place theory (sensu [16]) with various combinations of agriculture, specialized craft production (i.e., manufacturing), elite competition, and warfare driving hierarchical order among settlement systems by differentially dispersing and nucleating populations [11,17–23]. Given such behavioral models, we might expect hierarchical settlement patterns to be absent among hunter-gatherer societies, which often lack the complex socioeconomic drivers enumerated above.

Current studies of human settlement-size variation are overwhelmingly biased toward modern and historical settlement systems of western cultures [10,16,24–26], thus limiting our ability to evaluate the social and environmental contexts that foster the self-organization of hierarchical settlement structure. Considerable attention has been given to the structure of U.S. settlement systems, for example. The largest cities in U.S. settlement systems appear to exhibit power-law scaling in their size distribution [10,11,14,24,27]. Archaeological research extends the scope of settlement-size studies to include the settlement systems of prehistoric non-western cultures, especially state-organized societies such as Maya, Mesopotamia, and Tiwanaku [19,20,28,29]. To a lesser extent, non-state agricultural societies have also been examined [30,31]. Comparable analyses of settlement-size structure among hunter-gatherer societies are rare ostensibly because conventional wisdom holds that hierarchical structure of any form is antithetical to egalitarian hunter-gatherer economies. Yet, there is reason to suspect power-law structure in the absence of socioeconomic complexity. Some gregarious animal species appear to exhibit power-law scaling in their group sizes [32], and some predatory animals appear to exhibit power-law structure in the distribution of *waiting times—*the time spent in a given location before moving to another [33]. Analogous forms of either of these behaviors—differential aggregation or waiting times—could conceivably generate power-law structure in archaeological settlement-size variation in hunter-gatherer settlement systems.

Hamilton et al. [34] made the surprising observation that log-linear structure characterized group-size variation among 339 ethnographic hunter-gatherer settlement systems. Though the semi-quantitative data structure may or may not reflect power-law scaling per se, it is indicative of heavy-tailed structure and suggests the possibility that power-law structure is a property of hunter-gatherer group-size variation. Moreover, two other studies have argued that power-law scaling characterized waiting times in ethnographic! Kung foraging patterns [35,36]. While these novel studies represent key data points in our understanding of hunter-gatherer settlement structure, the fact that all ethnographic hunter-gatherers were economically integrated with sedentary agricultural and industrial societies to some degree [37,38] raises the concern that the observed scaling patterns are a result of those socioeconomic relations and not an endogenous property of hunter-gatherer systems.

Resolution of the question of power-law scaling in hunter-gatherer settlement size holds implications for our understanding of how complex socioeconomic structure self-organizes in human societies. The immediate goal of this analysis, then, is to reject the null hypothesis of power-law scaling among hunter-gatherer settlement systems. We analyze the statistical structure of settlement-size variation among eight prehistoric hunter-gatherer settlement systems that unequivocally existed in the absence of agricultural and industrial economies. We assume that if waiting times among hunter-gatherer settlements were power-law distributed, then artifact quantities among those sites should also be power-law distributed. If co-resident group size was power-law distributed, then both site area and artifact quantities should be power-law distributed.

The case studies examined here include 1779 temporally diagnostic artifacts from 405 archaeological sites. The total span of occupation for the sample includes more than 4000 years of the Early and Middle Holocene Epoch and three distinct environmental contexts including montane Peru, coastal Peru, and interior U.S. Southwest. While our analysis is able to reject the power-law hypothesis for site-area variation, it is unable to reject the power-law hypothesis for hunter-gatherer site-size variation as measured by artifact-per-site counts. Although this finding should not be construed as an assertion of power-law scaling, it strongly supports the presence of heavy-tailed statistical structure in the data and offers contingent support for power-law scaling. This support forms an independent and convergent line of empirical evidence that is immune to the biases faced by ethnographic data, albeit subject to its own biases. This paper describes the materials, methods, and results of the analysis. We conclude with a discussion of the study's implications for models of hunter-gatherer mobility and the self-organization of complexity in human societies.

## Materials and Methods

To test the hypothesis of power-law structure in hunter-gatherer settlement-size variation, we examine the size distribution of prehistoric archaeological sites in hunter-gatherer settlement systems. This section describes the sample, the archaeological proxies of settlement size, and the procedure used to test for power-law scaling.

### Archaeological Sample

**Fig 1:**
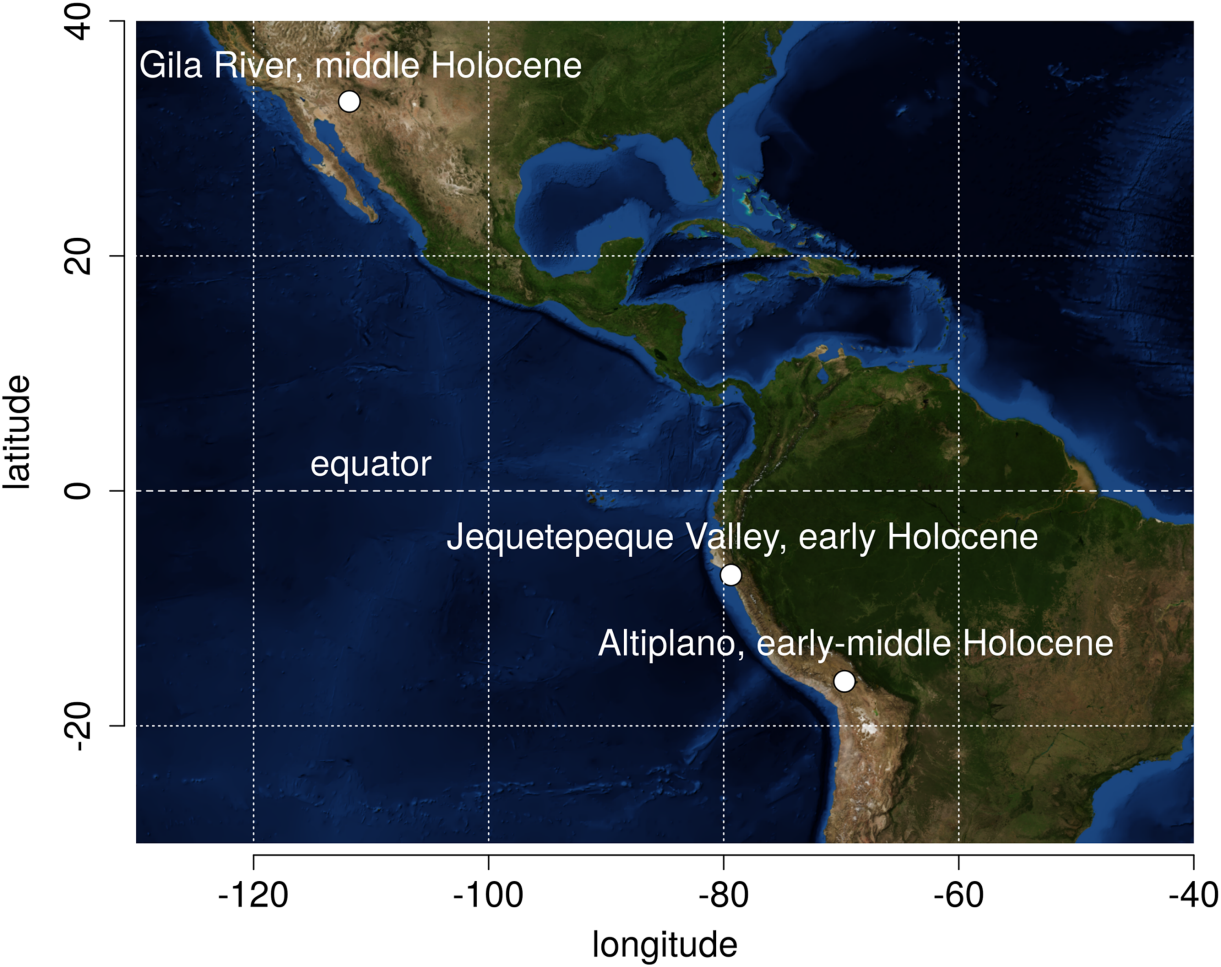
Geographic locations of prehistoric settlement systems examined in this study. Background image courtesy of NASA Earth Observatory [39].

**Fig 2:**
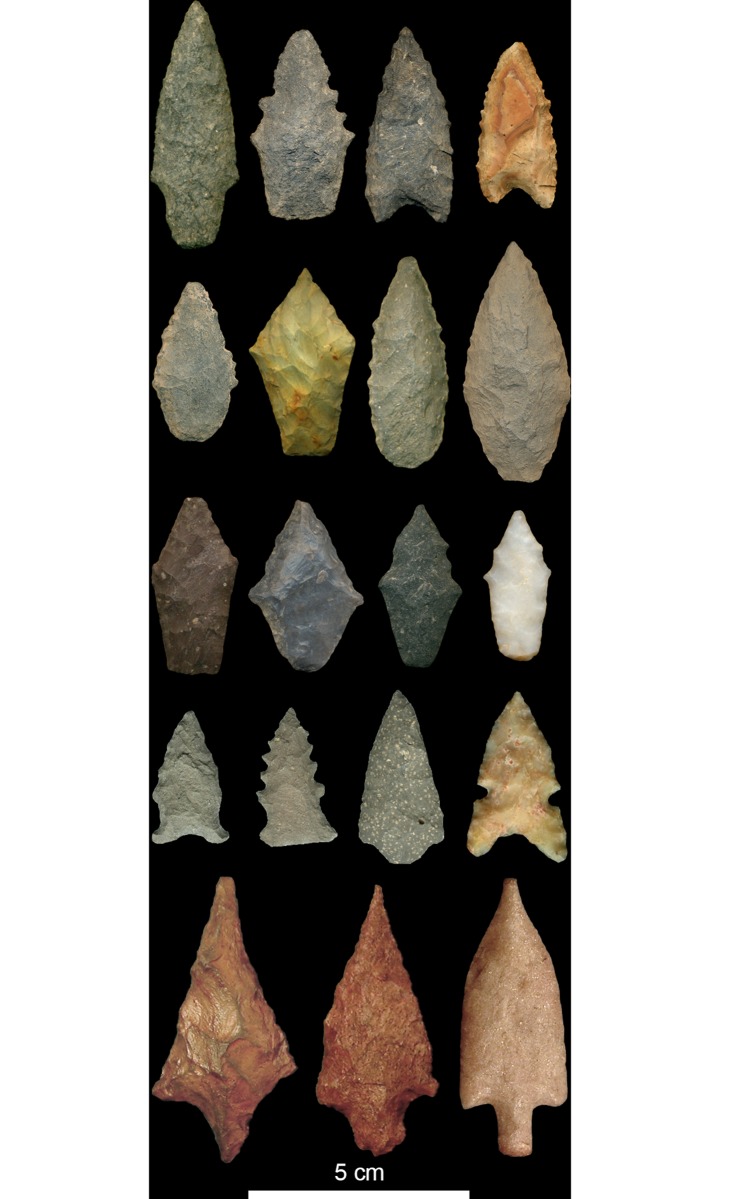
Examples of temporally diagnostic projectile points. Temporally diagnostic artifacts are used to (a) assign archaeological sites to settlement systems and (b) measure site size in terms of artifact counts. Top row: Titicaca Basin Late Archaic Period. 2^nd^ row: Titicaca Basin Middle Archaic Period. 3^rd^ row: Titicaca Basin Early Archaic Period. 4^th^ row: Gila River Middle Archaic (images reproduced with permission of author [43]). Bottom row: Jequetepeque Paijan.

The sample analyzed here consists of 1779 temporally diagnostic artifacts from 405 archaeological sites representing eight prehistoric New World settlement systems and three distinct arid environments (Figs 1 and 2, [39]). Each system represents an unequivocal hunter-gatherer economy marked by economic dependence on wild resources and high degree of residential mobility. Agricultural neighbors were either absent or highly unlikely in all cases. Environmental contexts range from 16° south latitude to 33° north latitude, sea level to over 3800 masl, seasonal to cold effective temperature regimes, arid to hyper-arid precipitation regimes, and desert to grassland biomes. The broad temporal and environmental scope of this sample serves to explore the generality of settlement-size structure within a narrow hunter-gatherer economic regime. For each settlement system, field researchers conducted systematic pedestrian surveys that recorded site locations and areal extents. Sites consist of spatially discrete artifact clusters separated by artifact-free expanses. One-hundred-percent surface collections of temporally diagnostic artifacts were conducted in each case. Artifact looting is absent or negligible in all cases, thus minimizing a potential source of sample bias. The surprising lack of projectile-point looting is attributable to cultural and historic circumstances specific to each region. In the Titicaca Basin, we have yet to observe an instance of avocational projectile-point collection during our combined experience in the region which spans over 30 years. In the Jequetepeque region, looters tend to overlook lithic artifacts in favor of gold and pottery from later contexts [40,41]. Sites on the Gila River Indian Reservation have remained relatively protected from collection due to cultural prohibitions against disturbing prehistoric sites [42].

The first environmental context considered is the western Lake Titicaca Basin in the Andes Mountains of highlands Peru. Elevations range from 3800 masl at Lake Titicaca to 6400 masl at the peak of Cerro Janq'u Uma. Human populations intensively inhabited the lower elevations in an environment known as the *Altiplano—*a vast expanse of rolling-hill grasslands dissected by perennial rivers and flanked by mountains [44]. Precipitation varies from approximately 300 to 900 mm/yr depending on elevation, local physiography, and climatic conditions. Mean daily temperature lows and highs range from -10°C to 19°C according to a seven-year period of record for the Inca Manco Cápac International Airport weather station in Juliaca, Peru.

Two study areas within the Titicaca Basin were examined. The first study area is the Río Ilave Basin, centered at 16°12'40”S, 69°43'20”W (WGS 1984). Elevations range from approximately 3830 to 3900 masl with adjacent mountains to 4600 masl. In a 41-km^2^ sample area of the Ilave Basin, Aldenderfer and colleagues recorded 90 archaeological sites with Archaic Period hunter-gatherer artifacts [45]. In addition, the first author revisited 24 of those sites and recorded 6 new sites. The second study area in the Titicaca Basin is the Río Huenque study area, centered at 16°45'50”S, 69°43'40”W (WGS 1984) [46]. The 33-km^2^ sample area occurs in a relatively restricted valley where elevations range between approximately 3940 and 4070 masl. Surrounding mountains rise to 5100 masl. Klink recorded 139 archaeological sites with hunter-gatherer artifacts in this area [46].

A detailed projectile point typology allows us to divide the long hunter-gatherer occupation of the Andean Altiplano into Early (11,500–9000 cal. B.P.), Middle (9000–7000 cal. B.P.), and Late (7000–5000 cal. B.P.) Archaic periods [47]. All of these periods represent subsistence economies reliant on vicuña (a wild camelid), taruca (Andean deer), wild seeds, and wild tubers [48]. The three temporal divisions in conjunction with the two environmental sub-contexts comprise six archaeological settlement systems.

The second environmental context considered is the Jequetepeque coastal plain and foothills of northern Peru, 1400 km northwest of the Altiplano study area. The extremely arid environment rarely receives more than 50 mm of rainfall per year. Mean daily temperature lows and highs range from 16°C to 31°C according to a 19-year period-of-record at the Capitán FAP José A. Quiñones Gonzales Airport weather station in Chiclayo, Peru. However, the Pacific littoral and lush alluvial plains offer highly productive, localized resource zones with diverse and often-abundant marine and terrestrial resources [49]. Dillehay and Maggard [50,51] conducted archaeological settlement surveys covering 70 km^2^ centered at 7°9'11"S, 79°22'31"W (WGS 84). The efforts recorded 126 hunter-gatherer sites with material evidence of *Paijan culture*—a hunter-gatherer tradition marked by distinctive flaked stone technologies that persisted from approximately 11,000–8500 cal. B.P. [51,52].

The third environmental context considered is located in the Middle Gila River of the U.S. Southwest at 33°8'53"N, 111°51'10"W, approximately 6000 km to the northwest of the Jequetepeque region. The Sonoran Desert environment averages 200 mm of precipitation per year. Mean daily temperature lows and highs range from 3°C to 40°C according to a 21-year period of record at the Casa Grande Municipal Airport weather station in Casa Grande, Arizona. Surface water is scarce and ephemeral. Major hunter-gatherer subsistence resources include bighorn sheep, whitetail deer, rabbit, mesquite seed pods, and cactus fruit [53]. Gila River Indian Community archaeologists reported Middle Archaic Period (5000–4000 cal. B.P.) projectile-point counts for 50 archaeological sites in a 591-km^2^ area [43]. These counts comprise the eighth and final case study investigated here.

Identification numbers for previously unpublished specimens are presented in S1 Specimens. All necessary permits were obtained for the described study, which complied with all relevant regulations. The archaeological specimens that inform this study were collected and curated in compliance with Peruvian law as stipulated in Resolución Directoral No 064-2013-DGPA-VMPCIC/MC issued to Haas, Aldenderfer, and Carlos Viviano Llave; DGPA-0122-2002 issued to Maggard and colleagues; and C038-98 issued to Klink by the Ministry of Culture, Republic of Peru. The Altiplano field collections are temporarily housed at the Collasuyo Archaeological Research Institute (Jr. Nicaragua 199, Puno, Puno, Peru) as stipulated in the respective research permits. The collections will be permanently curated at an official Ministry of Culture artifact repository to be determined at the time of dispossession. The Jequetepeque region collection is permanently curated at the Ministry of Culture's Huaca Arco Iris facility in Trujillo. No permits were required to use the previously published Gila River data.

### Measuring Settlement Size

We use two archaeologically tractable metrics of settlement size—artifact count and areal extent. It is first important to consider the possibility that the very act of defining sites might generate power-law structure in site-size variation (c.f., [54]). Defining sites is a subjective process in which field investigators identify artifact clusters, define their boundaries, and if necessary decide whether to split or aggregate adjacent clusters. In the study regions considered here, inter-cluster distances tend to be large relative to cluster size, thus distinguishing artifact clusters is relatively unambiguous in most instances. Regardless of the degree of ambiguity, we are unable to identify a clear theoretical link between the site-definition process and any of the generic mechanisms known to produce power-law or power-law-like structure [13,55]. We therefore currently have no theoretical reason to suspect that the site definition process poses a confounding factor in this study.

Given that sites represent behaviorally meaningful units of analysis, a site's artifact count is considered a relative proxy for person-hours of occupation, or cumulative waiting times. In general, the greater the number of individuals that occupy a settlement and the longer it is occupied, the greater the deposition of cultural materials. Thus, if hunter-gatherer waiting times were power-law distributed as previous studies have suggested [35,36], then we would expect to find that artifact-per-site counts are similarly distributed. To avoid over-estimation of artifact counts due to extraneous periods of activity, only temporally diagnostic artifacts for the periods of interest are used. Temporally diagnostic artifacts include projectile points in all eight settlement systems under consideration. In the Jequetepeque case, other temporally diagnostic tool types include bifacial preforms, scrapers, and *limaces*. Limaces are a unifacial, steep-edged flaked stone tools of uncertain function and are unique to Paijan culture [51]. In the north coast of Peru, bifacial flaked stone tools, scrapers, and limaces generally do not extend beyond the period of Paijan occupation and therefore can be considered diagnostic of that period. In highlands Peru and the U.S. Southwest, this is not the case. Thus, we are restricted to projectile points as temporally diagnostic artifacts in those cases. The raw artifact counts used in this analysis are provided in S1 Dataset.

A site's areal extent is considered a relative proxy for co-resident population size. In general, the more individuals that contemporaneously occupy a location, the more horizontal space they require. Thus, if co-resident group sizes were power-law distributed as previous studies have suggested [35,36], then we would expect to find that site-area values are similarly distributed [14,56]. Although specific activities may also affect site artifact counts and areas, we assume they are reasonable proxies for hunter-gatherer occupation intensity. This assumption finds empirical support in the ethnoarchaeological work of Yellen [57].

We compiled site-area estimates as reported by field analysts for seven of the eight settlement systems analyzed. Site-area estimates are not available for the Gila River case; however, as will be seen below, this omission does not affect the consistent results obtained in the other cases. In general, all of the field procedures for site-area estimation entailed qualitative identification of the maximal extents of artifact dispersion followed by maximal length and width estimations using tape or pace-based methods. Length and width dimensions were then multiplied to give area estimates.

Estimation of archaeological site area is potentially confounded by several sources of error. In this study, error sources include subjectivity in defining sites, imprecision in estimation of site dimensions, the use of length and width to estimate the areas of non-rectangular entities, and post-depositional movement of artifacts. Nonetheless, none of these factors are likely to confound this analysis because the error in each case tends to be linearly distributed and is thus insignificant relative to the question of non-linear structure in site-size variation. In other words, if site-area variation is truly power-law distributed, then such linear sources of error would be insufficient to bias the data to the extent that they would mask the extreme variation that non-linear power-law models entail. Moreover, the cumulative effects of such error sources would also be unlikely to confound tests of power-law structure. Such *multiplicative effects* are well known to generate lognormal variation as opposed to power-law variation [13,55].

Another confounding factor relates to reuse of sites by exogenous populations. Such occupations can inflate site-area estimates and therefore confound the settlement-area signal of the target system. We employ several bias-control measures to minimize this effect. For the Altiplano settlement systems, a site's area is included in a given dataset only if the majority of the diagnostic projectile points for that site can be assigned to the period of interest. In doing so, it is likely that the site's area is primarily a function of occupation during the period of interest. We consider three thresholds for inclusion in the Altiplano site-area datasets—greater than 50, 70, and 90 percent temporally diagnostic artifacts. Each threshold reflects a tradeoff between sample quality and size. Whereas higher thresholds offer more reliable samples, they tend to reduce the number of samples available for analysis. This sampling strategy produces 14 distinct datasets (two subregions x three time periods x three data thresholds—four equivalent pairs) for the Altiplano study area.

In the Jequetepeque case where flaked stone tool traditions are more constrained in time, we take three approaches to control for site-area inflation. Again, each approach reflects a tradeoff between sample quality and size. First, we examine all sites with one or more Paijan artifacts. Second, only sites identified as single component with Paijan artifacts are examined. Third, sites with one or more Paijan artifacts and excluding sites with ceramic artifacts are examined because ceramics are associated with later agricultural occupations. Site-area estimates are provided in S1 Dataset.

### Power-law Analysis of Settlement-Size Variation

Each dataset is analyzed in six steps in an effort to reject the hypothesis of power-law scaling. First, cumulative mass and cumulative density function (CMF and CDF, respectively) plots with logarithmic axes are used to inspect the data structure. Power-law distributions generate linear trends in such plots (i.e., they are log-linear) while other statistical structures tend to produce upwardly convex curves [13]. Second, we use maximum likelihood estimation (MLE) to find the best-fit model parameters for each of a candidate set of statistical models [27,58]. Because artifact-count data are discrete integer data, we consider Poisson, geometric, and discrete power-law distributions. For site-area data, which consist of continuous data measured in square meters, the candidate set of statistical models that we consider include normal, exponential, lognormal, and power-law (Pareto) distributions. Each of these statistical models considered has seen explicit or implicit use in the study of human settlement-size variation (e.g., [18,21,25]) and therefore merits consideration in our effort to reject the power-law hypothesis.

Third, we assess the statistical plausibility of each model fit to the data using the goodness-of-fit test described by Clauset et al. [27]. For each empirical dataset (*i*), consisting of *n*_*i*_ sites, we first solve for the KS distance between the empirical data and the MLE model (*D*_*m*_). Next, we draw a random sample of *n*_*i*_ values from the MLE-generated statistical model. We then solve for the KS distance between the empirical and synthetic data (*D*_*s*_). This procedure is then iterated 2500 times, and the fraction of times *D*_*s*_ is greater than *D*_*m*_ defines the probability (*p*) that the difference between the data and a given statistical model is a product of statistical chance alone. If *p* *≤* 0.10, then the model is rejected. If *p >* 0.10, then the model is considered plausible. The number of iterations and probability thresholds used here reflect the recommendations of Clauset et. al.

Fourth, we compare the relative information content of each statistically plausible model using Akaike information criterion (AIC) and AIC weights following the method described by Edward's et al. [59]. Models that generate low AIC weights (*w* ≤ 0.10) are rejected in favor of those that produce high AIC weights (*w* > 0.10).

Fifth, we consider power-law structure in the upper tails of the distributions. Unlike other statistical distributions, theoretical power-law distributions are scale invariant meaning that they obtain over an infinite range of values. Real-world phenomena, however, have finite size limits that restrict the potential range of applicability of power-law scaling [13]. Even in datasets where power-law models can be rejected for the full range of data, the possibility remains that power-law structure pertains to some upper-tail fraction of the data [13]. Defining this range is an analytical problem that must be addressed. To test for power-law scaling in the upper tails of the empirical distributions, we apply the iterative KS-test method of Clauset et al. [27] to find the most-probable threshold value (*x*_*min*_) for the hypothesized power-law tail of a given data sample. We then use MLE to find a best-fit power-law model for the tail and the previously described goodness-of-fit test to assess the statistical plausibility of the model. An upper-tail power-law model is rejected if the difference between the data and the model are unlikely to be explained by statistical chance (*p* *≤* 0.10).

Sixth and finally, we present a power analysis (not to be confused with *power-law* analysis), which serves two purposes. First, the power analysis serves to demonstrate that the methods and code function as intended. Second, the analysis serves to assess the probability of type I and II errors that might result given the sample size and statistical models under consideration. The power analysis consists of seven tests—one for each of the statistical models considered in this study. In each test, random samples are drawn from synthetic statistical distributions with known parameter values. The synthetic data are then analyzed with the same code used to analyze the empirical data. Sample sizes and parameter values for the synthetic data are selected at random from the set of sample sizes and parameter values observed in the empirical data. The procedure is iterated 100 times to evaluate the ratio of correct:incorrect model identifications. Correct identifications include those that produce insignificant *p* values (*p >* 0.1) and AIC weights (*w* > 0.1), indicating that the known model cannot be ruled out as providing a plausible fit to the data. Incorrect identifications include those that produce significant probability values (*p* *≤* 0.1) and AIC weights (*w* ≤ 0.1), indicating a poor fit between the model and the data despite the fact that their congruence is known. The ratio of correct:incorrect results gives a probabilistic measure of the procedure's efficacy, which we can then use to evaluate the robustness of the conclusions reached in the analysis of the empirical data. All calculations are performed using R statistical computing language including functions from MASS and PoweRlaw packages [60–62]. The code is presented in S1 Code.

## Results

**Fig 3:**
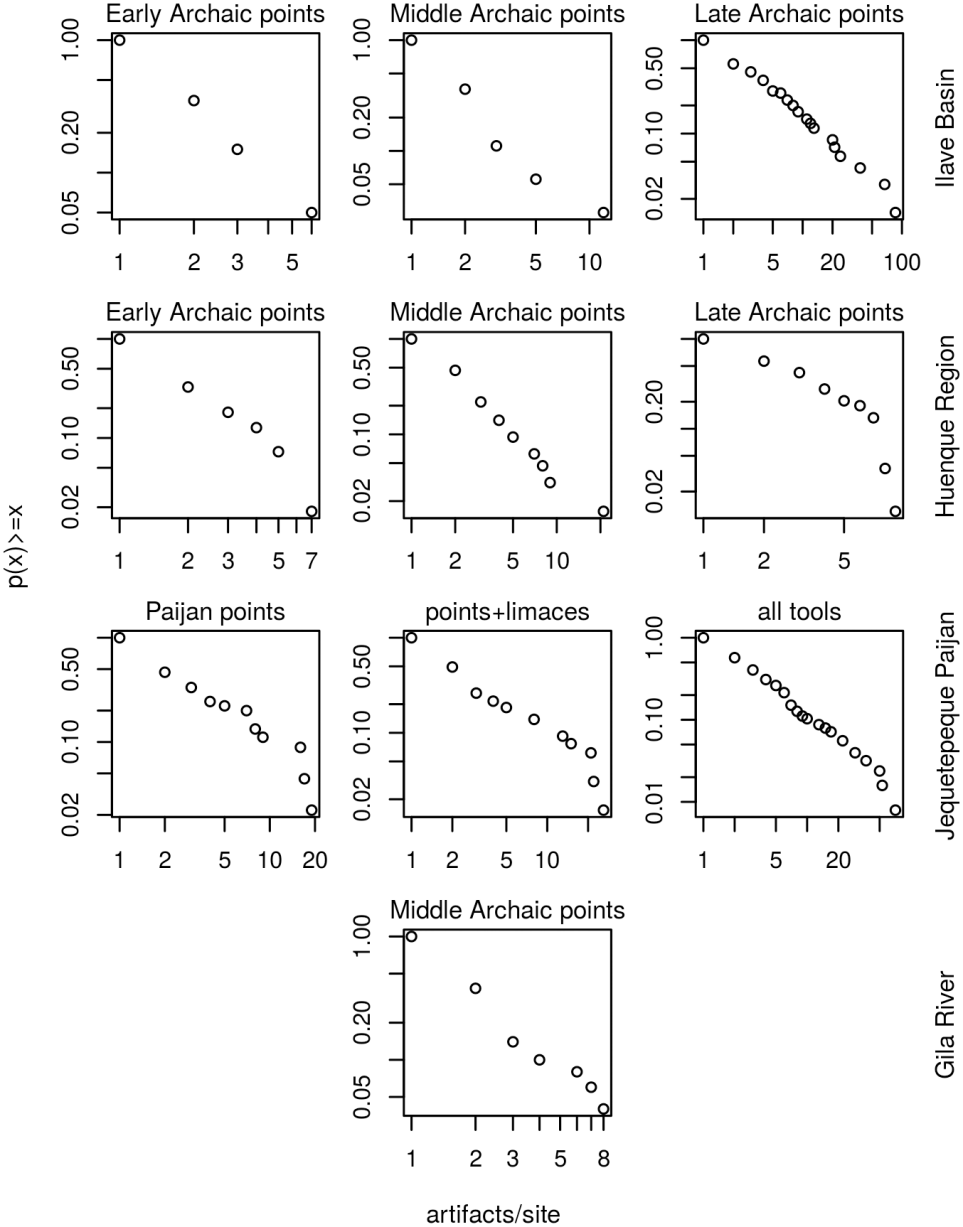
Cumulative mass function plots for artifact-per-site counts (discrete data). Axes are logarithmic. The log-linear structure is consistent with power-law structure.

**Fig 4:**
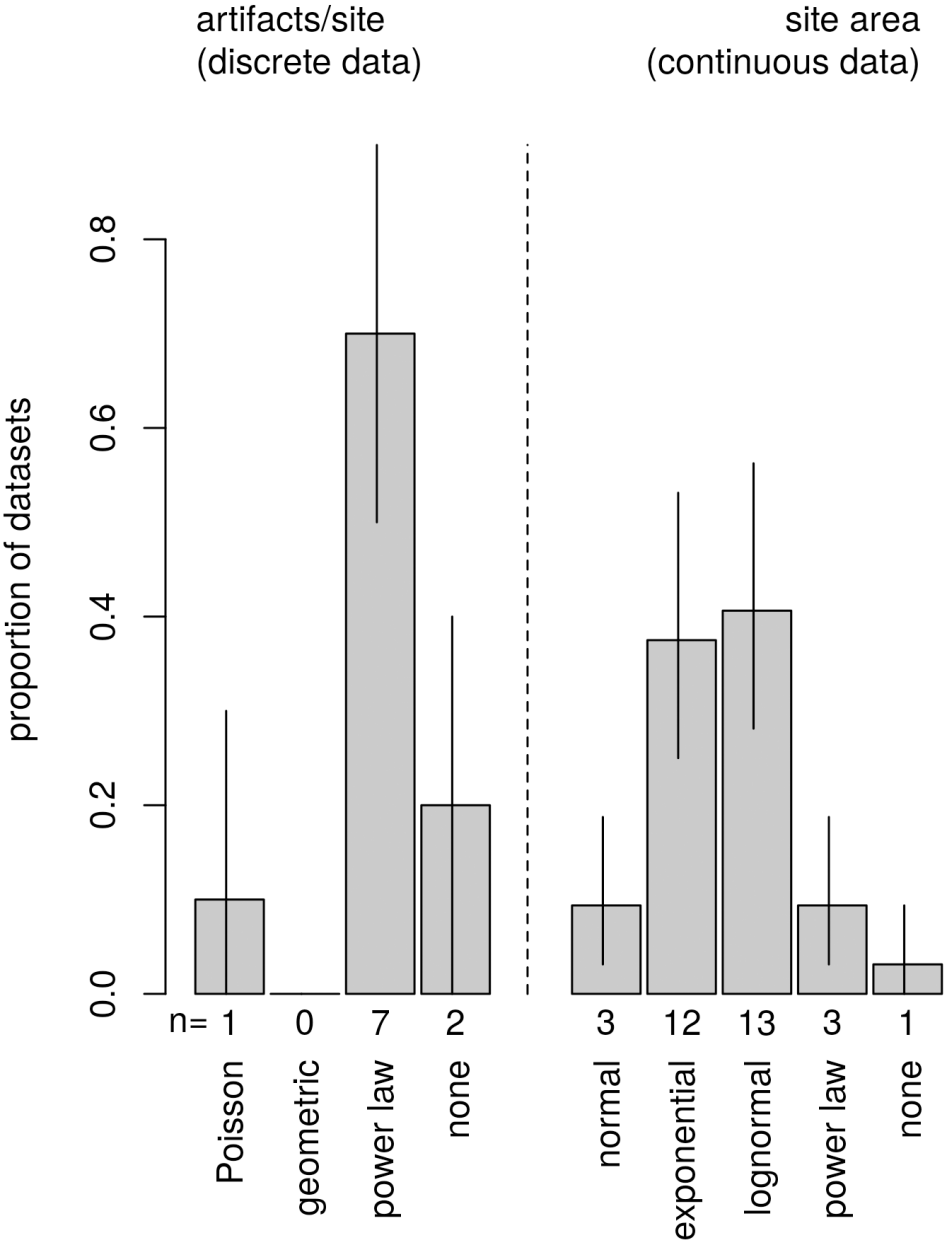
Summary of model-selection results for empirical datasets. Power-law models are favored in the artifact-count (discrete) data but not in the site-area (continuous) data. Bar labels indicate number of datasets found to fit the given statistical model. Proportions are within the discrete and continuous categories. Error bars indicate 5–95% quantile range derived by bootstrapping with 10,000 iterations.

For the discrete artifact-count data, CMF plots reveal clear log-linearity in all datasets suggesting power-law statistical structure (Fig 3). Maximum likelihood estimations of model parameters are presented in Table 1 along with the statistical plausibility results. The goodness-of-fit test is unable to reject power-law structure in seven of ten datasets (Fig 4). Conversely, best-fit Poisson models provide plausible fits to the data in just one of seven datasets, and geometric models offer implausible fits to all datasets. Two datasets did not produce plausible fits to any of the statistical models considered. Given that each dataset produced a single plausible result, AIC comparison is not applicable to the artifact count data.

**Table 1:** MLE parameters and goodness-of-fit results for artifact count data.

settlement system	dataset	artifacts	sites	statistical model	parameter estimates	KS D	*p*
Titicaca Basin Ilave Late Archaic	1. temporally diagnostic projectile points	463	70	Poisson	*λ* = 6.61	0.52	0.00
				geometric	*prob* = 0.13	0.25	0.00
				power law	*α* = 1.68, *x*_*min*_ = 1	0.08	0.11*
				power-law tail	*α* = 2.13, *x*_*min*_ = 6	0.07	0.62*
Titicaca Basin Huenque Late Archaic	2. temporally diagnostic projectile points	235	83	Poisson	*λ* = 2.83	0.23	0.81*
				geometric	*prob* = 0.26	0.45	0.00
				power law	*α* = 1.83, *x*_*min*_ = 1	0.12	0.01
				power-law tail	*α* = 10.75, *x*_*min*_ = 7	0.02	0.95^a^
Titicaca Basin Ilave Middle Archaic	3. temporally diagnostic projectile points	64	36	Poisson	*λ* = 1.78	0.47	0.00
				geometric	*prob* = 0.36	0.59	0.00
				power law	*α* = 2.35, *x*_*min*_ = 1	0.07	0.11*
				power-law tail	*α* = 3.08, *x*_*min*_ = 2	0.06	0.48^a^
Titicaca Basin Huenque Middle Archaic	4. temporally diagnostic projectile points	148	64	Poisson	*λ* = 2.31	0.33	0.07
				geometric	*prob* = 0.30	0.51	0.00
				power law	*α* = 2.06, *x*_*min*_ = 1	0.10	0.02
				power-law tail	*α* = 2.57, *x*_*min*_ = 2	0.03	0.97*
Titicaca Basin Ilave Early Archaic	5. temporally diagnostic projectile points	33	20	Poisson	*λ* = 1.65	0.51	0.01
				geometric	*prob* = 0.38	0.61	0.00
				power law	*α* = 2.37, *x*_*min*_ = 1	0.07	0.31*
				power-law tail	*α* = 2.37, *x*_*min*_ = 1	0.07	0.31*
Titicaca Basin Huenque Early Archaic	6. temporally diagnostic projectile points	96	55	Poisson	*λ* = 1.75	0.48	0.00
				geometric	*prob* = 0.36	0.60	0.00
				power law	*α* = 2.32, *x*_*min*_ = 1	0.04	0.53*
				power-law tail	*α* = 2.32, *x*_*min*_ = 1	0.04	0.53*
Gila River Middle Archaic	7. temporally diagnostic projectile points	94	50	Poisson	*λ* = 1.88	0.44	0.01
				geometric	*prob* = 0.35	0.57	0.00
				power law	*α* = 2.25, *x*_*min*_ = 1	0.07	0.14*
				power-law tail	*α* = 2.25, *x*_*min*_ = 1	0.07	0.14*
Jequetepeque Paijan	8. all flakestone tools	646	126	Poisson	*λ* = 5.13	0.48	0.00
				geometric	*prob* = 0.16	0.30	0.00
				power law	*α* = 1.73, *x*_*min*_ = 1	0.07	0.06
		490	33	power-law tail	*α* = 2.21, *x*_*min*_ = 5	0.07	0.36*
	9. Paijan points and limaces	237	65	Poisson	*λ* = 3.65	0.44	0.00
				geometric	*prob* = 0.22	0.38	0.00
				power law	*α* = 1.88, *x*_*min*_ = 1	0.05	0.34*
				power-law tail	*α* = 1.88, *x*_*min*_ = 1	0.05	0.34*
	10. Paijan points	163	45	Poisson	*λ* = 3.62	0.41	0.00
				geometric	*prob* = 0.22	0.39	0.00
				power law	*α* = 1.85, *x*_*min*_ = 1	0.07	0.34*
				power-law tail	*α* = 1.83, *x*_*min*_ = 1	0.07	0.34*

*Plausible models at p > 0.10.

^a^In some cases, the methods described above yield a p-value in the range of statistical plausibility but a power-law scaling parameter that exceeds the upper limit of acceptable values (*α* *≤* 3). Such values are theoretically problematic because they describe distributions that are not scale invariant and thus converge on non-power law distributions [15]. Moreover, such values are greater than those found to describe settlement hierarchy in the empirical cases of complex societies. For these reasons, an otherwise statistically plausible power-law model is rejected if the scaling parameter is greater than or equal to three.

**Fig 5:**
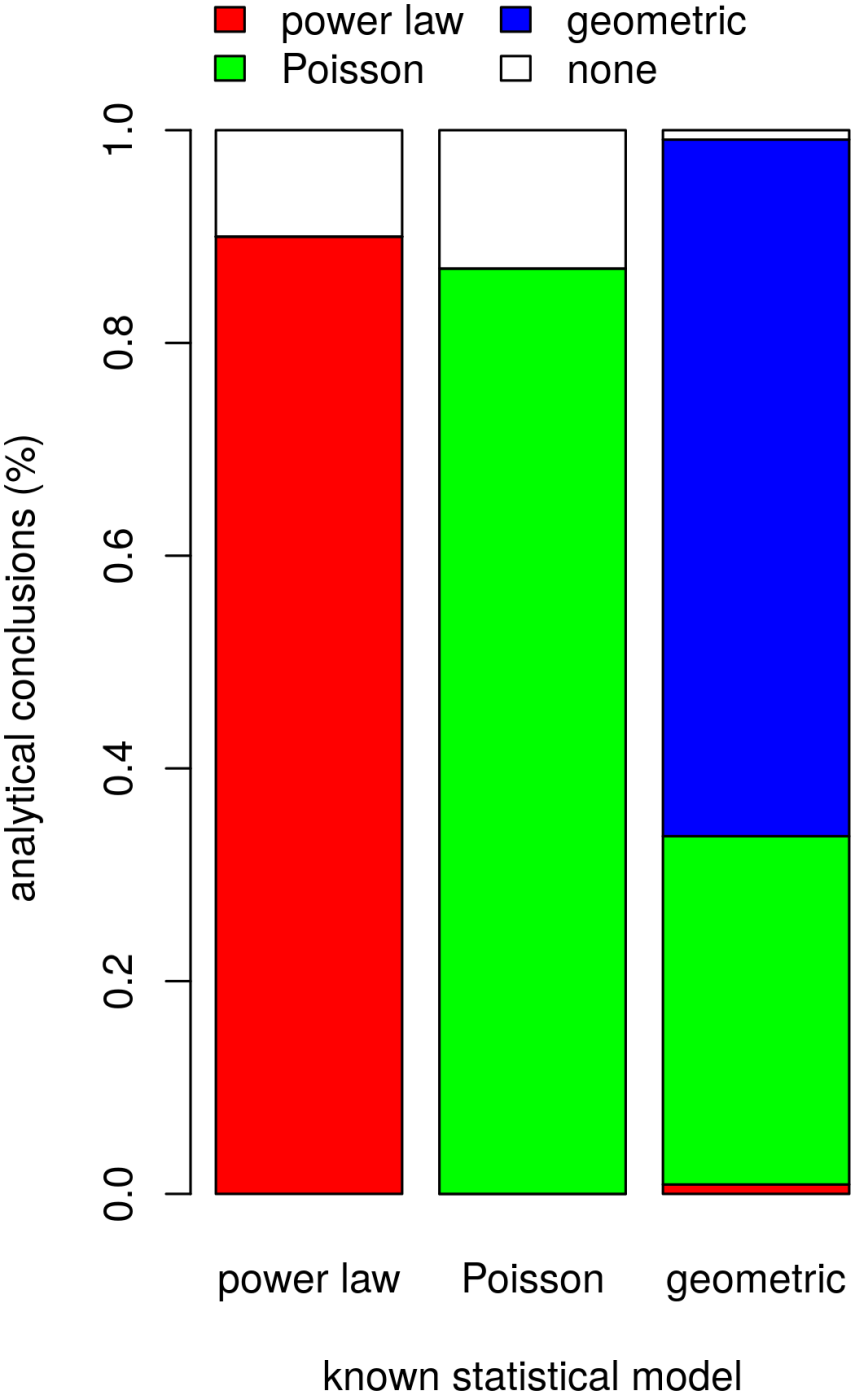
Results of power analysis for artifact-count (discrete) data. The analysis shows that given the sample sizes and MLE model-parameter values (a) the probability of failing to identify power-law structure when power-law structure is present (type II error) is highly unlikely and (b) the probability of spuriously identifying power-law structure given Poisson or geometric data (type I error) is also highly unlikely. See also S1 Table.

The power-analysis for the discrete artifact-count data confirms the method's efficacy and suggests an exceedingly small chance of obtaining the results by statistical chance alone. The procedure correctly identifies 90 percent of the synthetic power law data as consistent with power-law models (Fig 5, see also S1 Table). Power-law structure was never incorrectly identified given Poisson data and only once (1 percent) given geometric data. Accordingly, the power analysis results suggest an exceedingly small chance that the 7 datasets identified as consistent power-law models came from Poisson or geometric distributions. Moreover, the fact that 10 of the 100 of the synthetic power law models were misidentified (10 incorrect:90 correct) as inconsistent with all of the models under consideration raises the possibility that type II error could account for the 2 of 8 empirical datasets found to be inconsistent with all considered models (2 incorrect:6 correct; Fisher's Exact Test odds ratio = 0.34, 95% C.I. = 0.05–3.86, *p* = 0.22). In sum, the artifact-per-site datasets are generally consistent with power-law models and inconsistent with the alternatives.

**Fig 6:**
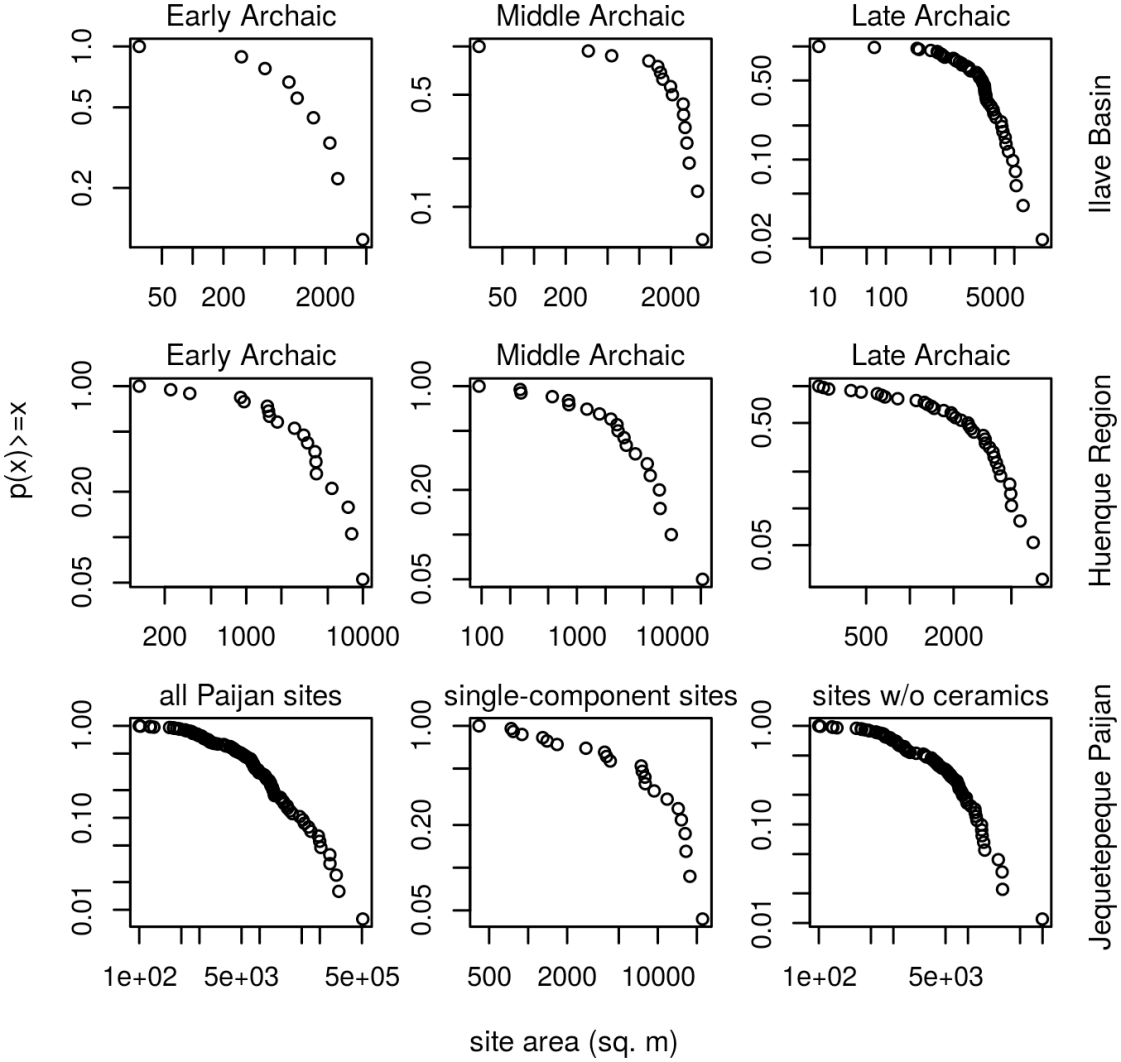
Cumulative density function plots for site-area (continuous) data. Axes are logarithmic. The upwardly convex data structure suggests an absence of power-law structure over the full range of data. Only the 50% threshold data are displayed for the Altiplano datasets.

For the continuous site-area data, CDF plots reveal convex structure over the full range of the data, suggesting an absence of power-law structure (Fig 6). Moreover, the more-rigorous goodness-of-fit and AIC analyses indicate that power law models offer poor characterizations of the full range of data in all seven archaeological settlement systems. Power law models are plausible and parsimonious for only three of the seventeen datasets, and other statistical models are also plausible in those three cases (Tables 2 and 3, see Fig 4). Normal distributions also offer plausible and parsimonious characterizations for just three datasets. In contrast, lognormal models are plausible and parsimonious for 13 of the 17 datasets, and exponential distributions are plausible and parsimonious for 12 of the 17 datasets. Only one dataset did not produce a plausible fit to any of the models considered. Power-law models offer plausible fits to the upper tails of 8 of the 17 datasets.

**Table 2:** MLE parameters and goodness-of-fit results for site area data.

settlement system	dataset	total site area (ha)	sites	statistical model	MLE parameter values	KS D	*p*
Titicaca Basin Ilave Late Archaic	1. sites > 50% Late Archaic points	20.3	51	normal	*μ* = 3983, *σ* = 4462	0.21	0.00
				exponential	*λ* = 2.51x10^-4^	0.08	0.72*
				lognormal	*μ* = 7.70, *σ* = 1.34	0.13	0.02
				power law	*α* = 1.18, *x*_*min*_ = 9	0.43	0.00
		11.3	11	power-law tail	*α* = 3.27, *x*_*min*_ = 6231	0.07	0.98^a^
	2. sites > 70% and > 90% Late Archaic points	15.4	33	normal	*μ* = 4658, *σ* = 5228	0.23	0.00
				exponential	*λ* = 2.15x10^-4^	0.11	0.61*
				lognormal	*μ* = 7.87, *σ* = 1.21	0.11	0.39*
				power law	*α* = 1.27, *x*_*min*_ = 66	0.37	0.00
		14.0	20	power-law tail	*α* = 2.27, *x*_*min*_ = 2574	0.14	0.12*
Titicaca Basin Huenque Late Archaic	3. sites > 50% Late Archaic points	10.0	38	normal	*μ* = 2637, *σ* = 1919	0.11	0.35*
				exponential	*λ* = 3.79x10^-4^	0.12	0.35*
				lognormal	*μ* = 7.53, *σ* = 0.94	0.13	0.14*
				power law	*α* = 1.47, *x*_*min*_ = 236	0.29	0.00
		6.9	15	power-law tail	*α* = 3.91, *x*_*min*_ = 3204	0.13	0.48^a^
	4. sites > 70% Late Archaic points	6.0	23	normal	*μ* = 2591, *σ* = 1864	0.12	0.55*
				exponential	*λ* = 3.86x10^-4^	0.14	0.45*
				lognormal	*μ* = 7.51, *σ* = 0.94	0.16	0.13*
				power law	*α* = 1.49, *x*_*min*_ = 236	0.28	0.01
		4.4	10	power-law tail	*α* = 4.45, *x*_*min*_ = 3204	0.16	0.39^a^
	5. sites > 90% Late Archaic points	2.9	14	normal	*μ* = 2083, *σ* = 1983	0.19	0.17*
				exponential	*λ* = 4.80x10^-4^	0.15	0.72*
				lognormal	*μ* = 7.17, *σ* = 1.01	0.17	0.33*
				power law	*α* = 1.59, *x*_*min*_ = 236	0.21	0.18*
		2.9	13	power-law tail	*α* = 1.76, *x*_*min*_ = 393	0.17	0.15*
Titicaca Basin Ilave Middle Archaic	6. sites > 50% Middle Archaic points	3.2	16	normal	*μ* = 2017, *σ* = 1098	0.14	0.54*
				exponential	*λ* = 4.96x10^-4^	0.27	0.03
				lognormal	*μ* = 7.25, *σ* = 1.18	0.16	0.00
				power law	*α* = 1.24, *x*_*min*_ = 30	0.41	0.01
		1.9	6	power-law tail	*α* = 6.25, *x*_*min*_ = 2632	0.17	0.50^a^
	7. Sites > 70% and > 90% Early Archaic Points	1.5	8	normal	*μ* = 1884, *σ* = 906	0.22	0.36*
				exponential	*λ* = 5.3x10^-4^	0.35	0.05
				lognormal	*μ* = 7.41, *σ* = 0.54	0.18	0.70*
				power law	*α* = 1.90, *x*_*min*_ = 546	0.39	0.02
		1.3	6	power-law tail	*α* = 4.00, *x*_*min*_ = 1500	0.21	0.43^a^
Titicaca Basin Huenque Middle Archaic	8. sites > 50% Middle Archaic points	8.0	20	normal	*μ* = 4040, *σ* = 4702	0.21	0.02
				exponential	*λ* = 2.48x10^-4^	0.12	0.84*
				lognormal	*μ* = 7.61, *σ* = 1.34	0.14	0.43*
				power law	*α* = 1.31, *x*_*min*_ = 94	0.29	0.02
		5.7	6	power-law tail	*α* = 2.98, *x*_*min*_ = 5479	0.10	0.87*
	9. sites > 70% and > 90% Middle Archaic points	3.8	8	normal	*μ* = 4757, *σ* = 6485	0.31	0.04
				exponential	*λ* = 2.10x10^-4^	0.34	0.06
				lognormal	*μ* = 7.33, *σ* = 1.72	0.22	0.35*
				power law	*α* = 1.36, *x*_*min*_ = 94	0.24	0.41*
		3.6	4	power-law tail	*α* = 2.76, *x*_*min*_ = 4109	0.19	0.81*
Titicaca Basin Ilave Early Archaic	10. sites > 50% Early Archaic points	1.4	9	normal	*μ* = 1527, *σ* = 1362	0.19	0.48*
				exponential	*λ* = 6.55x10^-4^	0.10	1.00*
				lognormal	*μ* = 6.71, *σ* = 1.41	0.18	0.55*
				power law	*α* = 1.27, *x*_*min*_ = 30	0.35	0.08
		0.9	3	power-law tail	*α* = 3.17, *x*_*min*_ = 2201	0.13	0.71^a^
	11. sites > 70% and > 90% Early Archaic Points	0.7	3	normal	*μ* = 2375, *σ* = 1769	0.23	0.86*
				exponential	*λ* = 4.21x10^-4^	0.27	0.85*
				lognormal	*μ* = 7.28, *σ* = 1.15	0.31	0.50*
				power law	*α* = 1.63, *x*_*min*_ = 300	0.38	0.30*
				power-law tail	*α* = 1.63, *x*_*min*_ = 300	0.38	0.30*
Titicaca Basin Huenque Early Archaic	12. sites > 50% Early Archaic points	6.1	19	normal	*μ* = 3200, *σ* = 2727	0.18	0.12*
				exponential	*λ* = 3.12x10^-4^	0.11	0.87*
				lognormal	*μ* = 7.57, *σ* = 1.18	0.15	0.27*
				power law	*α* = 1.34, *x*_*min*_ = 121	0.34	0.00
		2.5	3	power-law tail	*α* = 6.60, *x*_*min*_ = 7477	0.13	0.65^a^
	13. sites > 70% Early Archaic points	3.7	13	normal	*μ* = 2883, *σ* = 2933	0.21	0.11*
				exponential	*λ* = 3.47x10^-4^	0.12	0.94*
				lognormal	*μ* = 7.31, *σ* = 1.30	0.12	0.86*
				power law	*α* = 1.40, *x*_*min*_ = 121	0.32	0.00
		0.3	6	power-law tail	*α* = 2.67, *x*_*min*_ = 2592	0.18	0.49*
	14. sites > 90% Early Archaic points	0.3	12	normal	*μ* = 2907, *σ* = 3051	0.25	0.04
				exponential	*λ* = 3.44x10^-4^	0.17	0.67*
				lognormal	*μ* = 7.27, *σ* = 1.34	0.15	0.63*
				power law	*α* = 1.40, *x*_*min*_ = 121	0.30	0.00
		0.3	9	power-law tail	*α* = 1.90, *x*_*min*_ = 895	0.25	0.02
Jequetepeque Paijan	15. all sites with one or more Paijan-diagnostic artifacts	254.6	126	normal	*μ* = 20205, *σ* = 55992	0.36	0.00
				exponential	*λ* = 4.95x10^-5^	0.32	0.00
				lognormal	*μ* = 8.39, *σ* = 1.73	0.08	0.08
				power law	*α* = 1.26, *x*_*min*_ = 100	0.28	0.98^a^
		238.7	53	power-law tail	*α* = 1.87, *x*_*min*_ = 7209	0.06	0.97*
	16. excluding sites with non-Paijan artifacts	17.6	23	normal	*μ* = 7674, *σ* = 6447	0.18	0.05
				exponential	*λ* = 1.30x10^-4^	0.14	0.47*
				lognormal	*μ* = 8.41, *σ* = 1.18	0.19	0.03
				power law	*α* = 1.40, *x*_*min*_ = 418	0.24	0.05
		8.8	5	power-law tail	*α* = 6.91, *x*_*min*_ = 15200	0.13	0.62^a^
	17. excluding sites with ceramics	54.6	91	normal	*μ* = 6005, *σ* = 11561	0.30	0.00
				exponential	*λ* = 1.67x10^-4^	0.23	0.00
				lognormal	*μ* = 7.79, *σ* = 1.40	0.08	0.15*
				power law	*α* = 1.31, *x*_*min*_ = 100	0.29	0.00
		42.7	27	power-law tail	*α* = 2.42, *x*_*min*_ = 6250	0.10	0.32*

* Plausible models at p > 0.10.

^a^In some cases, the methods described above yield a p-value in the range of statistical plausibility but a power-law scaling parameter that exceeds the upper limit of acceptable values (*α* *≤* 3). Such values are theoretically problematic because they describe distributions that are not scale invariant and thus converge on non-power law distributions [15]. Moreover, such values are greater than those found to describe settlement hierarchy in the empirical cases of complex societies. For these reasons, an otherwise statistically plausible power-law model is rejected if the scaling parameter is greater than or equal to three.

**Table 3:** Results of AIC and AIC weight analysis.

case	metric	constraints	plausible statistical models	AIC	AIC weight
Ilave Late Archaic	site area	sites > 70% Late Archaic points	exponential	626	0.89*
			lognormal	630	0.11*
Rio Huenque Late Archaic	site area	sites > 50% Late Archaic points	normal	686	0.06
			exponential	677	0.63*
			lognormal	680	0.31*
		sites > 70% Late Archaic points	normal	416	0.08
			exponential	410	0.61*
			lognormal	412	0.31*
		sites > 90% Late Archaic points	normal	256	0.02
			exponential	244	0.39*
			lognormal	245	0.33*
			power law	246	0.26*
Ilave Basin Middle Archaic	site area	sites > 70% and > 90%Middle Archaic points	normal	135.6	0.46*
			lognormal	135.3	0.54*
Rio Huenque Middle Archaic	site area	sites > 50% Middle Archaic points	exponential	374	0.72*
			lognormal	377	0.28*
		sites > 70% and > 90% Middle Archaic points	lognormal	153	0.38*
			power law	152	0.62*
Ilave Basin Early Archaic	site area	sites > 50% Early Archaic points	normal	159	0.06
			exponential	152	0.77*
			lognormal	157	0.17*
		sites > 70% and > 90%Early Archaic points	normal	57.5	0.16*
			exponential	55.6	0.32*
			lognormal	57.1	0.18*
			power law	54.4	0.34*
Rio Huenque Early Archaic	site area	sites > 50% Early Archaic points	normal	359	0.04
			exponential	347	0.75*
			lognormal	352	0.21*
		sites > 70% Early Archaic points	normal	249	0.01
			exponential	235	0.71*
			lognormal	238	0.28*
		sites > 90% Early Archaic points	exponential	217	0.75*
			lognormal	220	0.25*

*Favored results at w > 0.10. The analysis only includes cases where two or more statistical models were found to be statistically plausible (see Table 2). Multiple plausible models were not observed in the discrete data cases (see Table 1) and are therefore not included in this table.

**Fig 7:**
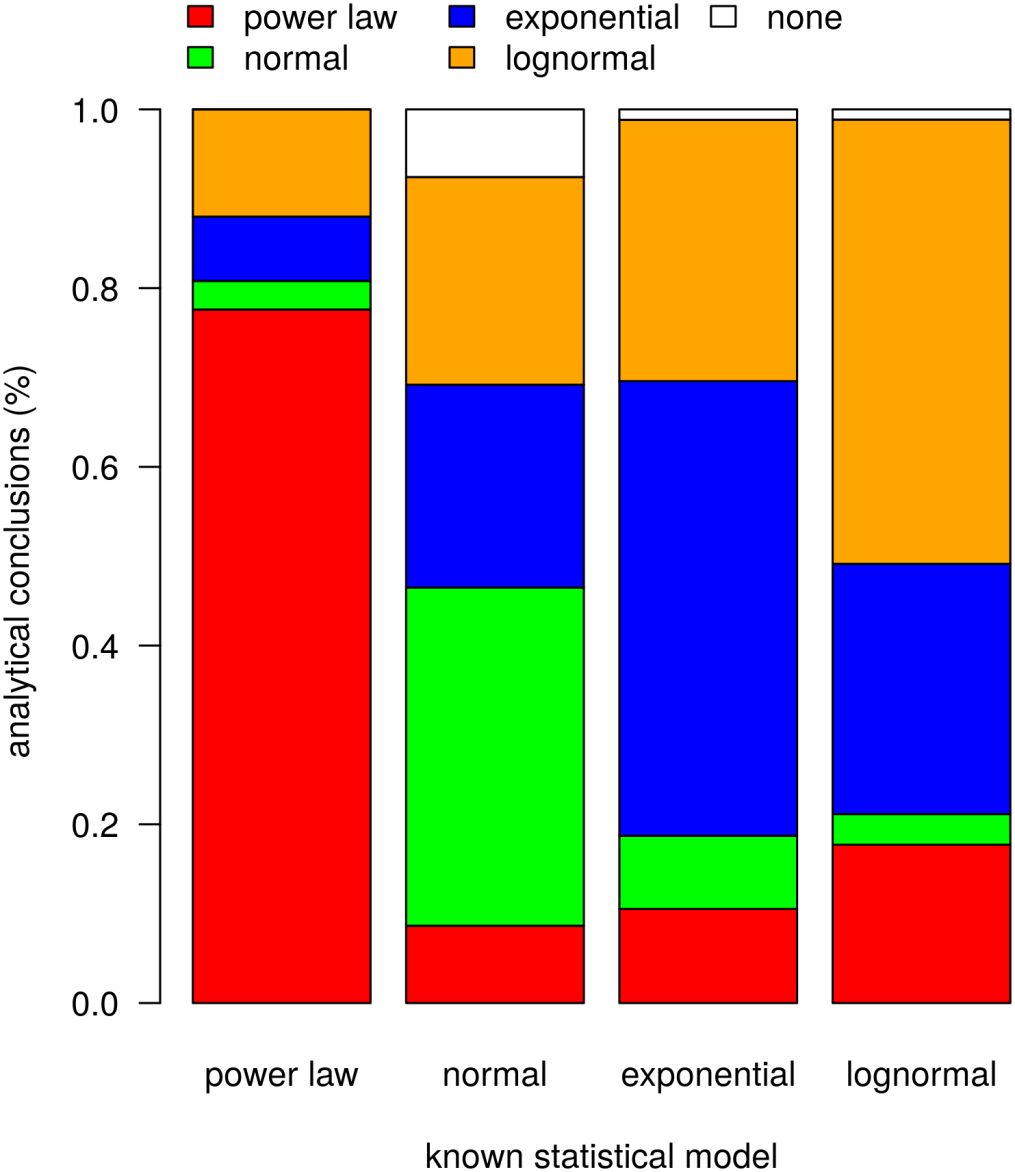
Results of power analysis for site-area (continuous) data. The analysis shows that given the sample sizes and MLE model-parameter values, (a) the probability of failing to identify power-law structure when power-law structure is present (type II error) is unlikely and (b) the probability of spuriously identifying power-law structure given normal, exponential, or lognormal data (type I error) is also unlikely. See also S2 Table.

The power analysis results for the continuous data are presented in Fig 7 (see also S2 Table). The results confirm the method's efficacy and suggest an exceedingly small chance of obtaining the analytical results by statistical chance alone. Given samples drawn from power-law models with parameter values in the range of the empirically estimated values, the procedure correctly identifies 97 percent as consistent with power-law models. Moreover, power-law structure is identified in the upper tails of 97 percent of the synthetic power-law data samples.

However, the procedure also incorrectly identifies models with low frequency. Because the empirical data show that power-law structure is highly unlikely to obtain over the full range of empirical data but is plausible for the upper tails of the data, we are most concerned here with how likely power-law structure is to be spuriously identified in the upper tails of non-power-law samples. The procedure incorrectly finds power-law structure in the upper tails of 67 percent of the lognormal data samples. The same misidentification occurs 27 percent of the time given exponential data. Normally distributed data generate upper tails that are spuriously identified as power-law distributed 9 percent of the time.

Recall that the archaeological site-area data produced 8 of 17 datasets with plausible power-law structure in the upper tails of the distributions. Given that (a) exponential, lognormal, and normal structure is found to be consistent for the full range of data 12, 13 and 3 times, respectively, in the 17 continuous archaeological datasets and (b) the proportion of times we expect those distributions to generate upper tails identifiable as power-law distributed, we would expect to have spuriously identified power-law structure in the upper tails in 0.27 * 12 + 0.67 * 13 +0.09 * 3 = 12 of 17 datasets. This expectation more than accounts for the 8 of 17 archaeological datasets with plausible power-law tails, leading us to conclude that in the case of site-area data, the observed plausibility of power-law structure in the upper tails may simply be an artifact of sample uncertainty.

In sum, the results show that power-law models provide plausible and parsimonious characterizations of hunter-gatherer site-size variation when settlement-size is measured by artifact counts but that power-law models are unlikely to characterize hunter-gatherer settlement-size variation when settlement size is measured by areal extent.

## Discussion

On one hand, previous research on agricultural and state-organized societies has suggested that power-law scaling of settlement-size variation is a property of complex, hierarchical socioeconomic processes, which tend to be absent among hunter-gatherer societies. On the other hand, ethnographic research has suggested that power-law scaling characterized hunter-gatherer settlement-size variation. Although the latter claim would seem to trump the former, ethnographic data limitations cast some concern on the degree to which scaling properties are intrinsic to hunter-gatherer systems or are artifacts of economic connections with sedentary societies. To our knowledge, this paper presents the first rigorous analysis of hunter-gatherer settlement-size variation as observed through archaeological data. The analysis provides an independent, complementary test that is immune to the limitations faced by ethnographic observations, albeit with its own limitations.

We have reasoned that if hunter-gatherer waiting times varied as a power-law function, then artifact-per-site quantities in a given hunter-gatherer settlement system should also vary as a power-law function. If hunter-gatherer group-size varied as a power-law function, then both site areas and artifact quantities in a given hunter-gatherer settlement system should vary as a power-law function. Although the analysis rejected power-law scaling for site-area variation, it was unable to reject power-law scaling for hunter-gatherer site-size variation as measured by artifact-per-site counts. These conclusions are consistent with a model of power-law distributed site occupation spans, or cumulative waiting times, and inconsistent with a model of power-law distributed co-resident group sizes.

To be sure, our inability to reject power-law scaling in artifact-count variation should not be confused with assertion of power-law structure. Alternative statistical models may yet offer stronger fit to the data and should be explored as theory dictates. Regardless, it is clear that hunter-gatherer site-size variation in arid environments tends to exhibit heavy-tailed structure. Hunter-gatherer research now faces the challenge of explaining the structural properties described here. We currently lack models of hunter-gatherer mobility, social interaction, or site formation that explicitly predict this structure. Efficacious models will be those that predict (a) heavy-tailed statistical structure in the size-distributions of hunter-gatherer settlements as measured by cumulative occupation time and (b) exponential or lognormal structure as measured by co-resident group size.

### A Preferential Attachment Model of Forager Mobility

We briefly consider a candidate model here to offer a potential guide for future research. The working model posits that heavy-tailed site-size variation in hunter-gatherer settlement systems was a property of long-term *preferential attachment* to places on landscapes. Preferential attachment is a term that statistical physicists use to describe a generic class of processes that entail feedback loops [13,55]. Importantly, preferential attachment is one of several mechanisms known to give rise to power-law structure. The “rich-get-richer” is a classic example of a preferential attachment process that gives rise extreme wealth disparity among individuals in a given society and is often characterized by power-law (i.e., Pareto) models [13]. We can readily imagine that preferential attachment to places on landscapes played an important role in hunter-gatherer residential mobility decisions thus giving rise to heavy-tailed variation in the differential accumulation of site occupation times and thus artifacts. Anthropologists have suggested a variety of mechanisms by which humans become “attached” to places, including economic and symbolic mechanisms [63–67]. We might therefore imagine that as hunter-gatherers moved across landscapes to take advantage of seasonally available resources, they preferentially reoccupied certain locations to access previously discarded materials, cultural infrastructure, or cultural meaning. While most settlements would have experienced modest, short-term occupation and material accumulation, some locations would have experienced compounding occupation intensity that would have driven extreme material accumulation over the long-term.

Importantly, this model does not predict power-law scaling in site-area variation and is thus consistent with the rejection of power-law scaling in the empirical analysis presented here. We might further consider the type of site-area variation that this preferential attachment model does predict. As a first approximation, the range of variation is expected to be more constrained than that of power-law variation. This is because temporally distributed reoccupations of locations entail some degree of spatial overlap thus limiting a site's areal growth rate relative to its quantitative growth rate (i.e., artifact accumulation). Of the continuous statistical distributions considered in this analysis, normal, exponential, and lognormal models fit this general expectation of comparatively low dispersion. Furthermore, we can rule out normal distribution models given that site areas cannot be negative by definition. To decide between exponential and lognormal models, we consider site-formation processes in light of the generic processes known to give rise to exponential and lognormal structure. Site area is expected to vary as a function of co-resident group size, spatial non-overlap between sequential occupations, and the spatial dispersion of cultural materials in systemic and taphonomic contexts. Without additional theoretical guidance as to which of these factors are most important, we might simply consider a null expectation in which each variable randomly contributes to site-area variation. Because lognormal distributions are the product of many independent random events [55], we suggest lognormal site-area variation could be expected under the model of preferential attachment. Given the empirical results of this study, we currently cannot rule out this expectation of the preferential attachment model. However, it is important to note that exponential models also present plausible fits to the empirical data. Additional research is needed to determine which statistical model offers a better fit and whether or not there is a theoretical basis for exponential variation in site areas.

### Implications of the Working Model

The preferential attachment model of forager mobility holds a number of anthropological implications, and we briefly consider several here including implications for archaeological site formation, settlement-size variation in diverse environmental contexts, and self-organization of settlement-size hierarchies in human societies. Regarding the structure of site formation, the model suggests that artifact accumulation among the sites of a given hunter-gatherer settlement system would have been distributed through time such that that largest sites would be expected to exhibit occupation spans that approach the temporal span of the settlement system's existence. For many archaeologically visible hunter-gatherer systems, such spans may be on the order of centuries to millennia. This expectation follows from the assumption of preferential attachment, which implies that the attractiveness of a site is partially a function of the intensity of previous occupations. Thus, even a chance resource encounter at some otherwise unexceptional location on the landscape could lock hunter-gatherers into persistent reoccupation over the long-term. Importantly, such long-term uses of highly localized sites can be expected even in the absence of highly localized natural resources such as caves, rockshelters, or springs. We should therefore expect chronological analyses of large open-air hunter-gatherer sites to produce decadal- to millennial-scale occupation spans even in the absence of residential sedentism or spatially localized natural resources.

The preferential attachment model also holds implications for settlement-size variation across environmental contexts. First, because all anatomically modern hunter-gatherers relied on material and symbolic culture, we should expect that preferential attachment to places applied to all hunter-gatherers in all environmental contexts. Thus, we should expect to observe heavy-tailed settlement-size variation across environmental contexts. Nonetheless, the center of mass of site-size variation should vary across environmental contexts. Resource richness should negatively predict the strength of preferential attachment and thus the scaling exponent of power-law models for a given set of settlement systems. This is because resource poor environments would tend to exert greater pressure on resource recycling behavior and thus the reocupation of sites.

Last, the preferential attachment model offers potential insights into how settlement-size hierarchies may have self-organized in human societies through a common behavioral process that transcends economic extremes. When populations were low, as is often the case for hunter-gatherer societies, co-resident population size at the largest settlements would have been restricted, thus limiting site growth in areal extent relative to growth in material accumulation. In other words, for low-density hunter-gatherer populations, settlement-size scaling would have been a property of preferential attachment among settlements with largely *asynchronous* occupation. Conversely, preferential attachment could be expected to generate different site-formation results when population densities are high, as is often the case for agrarian and industrial societies. When individuals make residential moves in these cases, albeit with lower frequency than hunter-gatherers, preferential attachment to places would tend to result in multi-resident, multi-family occupations of settlements. These co-resident populations would have required a proportionate amount of space, thus material accumulations would have expanded spatially at a rate that was commensurate with the rate of quantitative accumulation. For high-density populations, settlement-size scaling would therefore have been a property of preferential attachment among settlements with largely *synchronous* occupation, as is the case among most modern societies.

To the extent that this preferential attachment model is viable, it would help us understand the similarities and differences in settlement-size variation observed in mobile and sedentary societies. In turn, it tentatively suggests a trajectory for the self-organization of settlement-size hierarchies in human societies. As hunter-gatherer populations grew, land use intensified, and residential mobility decreased, asynchronous settlement-size scaling structure would have gradually given way to the synchronous scaling structure that characterizes settlement-size hierarchies in settled agrarian societies. If so, incipient forms of hunter-gatherer settlement-size hierarchy would have created a context for the self-organization of socioeconomic complexity including the hierarchical structure of political and economic organization. This cultural trajectory differs from previous thinking, which has tended to see settlement-size hierarchy as a distinctive feature of hierarchical, state-organized, and industrial societies [17,22,23]. The model proposed here does not undermine previous models that use complex socioeconomic behaviors such as agriculture, manufacturing, and warfare to explain settlement-size hierarchy (e.g., [11,19,68]), but it does suggest that such complex behaviors may be proximate to more fundamental socioeconomic behaviors that existed among states and hunter-gatherer societies alike (see also [34] for a similar conclusion). We suggest that the shared behavior spanning economic extremes may be residential mobility guided by preferential attachment to places.

In conclusion, this study found that prehistoric hunter-gatherer settlement systems of the New World exhibit heavy-tailed statistical structure that is consistent with power-law scaling. We have interpreted this archaeological variation to reflect extreme variation in the occupation spans of hunter-gatherer sites. We speculate that the statistical structure may have been a self-organized property of preferential attachment behavior whereby foraging populations preferentially occupied certain locations on the landscape to take advantage of material culture or symbolic resources. In turn, the behavior and its macro-scale outcomes may have laid a structural foundation for self-organized settlement hierarchies in subsequent times. We emphasize that this working model simply suggests an analytical starting point. Alternative models such as post-depositional process models (e.g., [69]) require additional consideration. We hope that these analytical findings and theoretical considerations stimulate additional efforts to link hunter-gatherer behavior to the observed structural properties of human settlement patterns—patterns that fundamentally shape the organization and dynamics of human societies.

## Supporting Information

S1 Specimens Archaeological specimens. (CSV)

S1 Dataset Artifact count and site area data. (TXT)

S1 Code R code used to analyze data. (ZIP)

S1 Table Numerical results of power analysis for artifact-count data. (PDF)

S2 Table Numerical results of power analysis for site-area data. (PDF)
