# Supplementary material for: Settlement-Size Scaling among Prehistoric Hunter-Gatherer Settlement Systems in the New World
Source: PLoS One. 2015 Nov 4;10(11):e0140127. doi: 10.1371/journal.pone.0140127 (PMC4633060; doi:10.1371/journal.pone.0140127)
Supplement: S1 Table — (PDF) [file pone.0140127.s004.pdf]

S1 Table. Numerical results of power analysis for artifact-count data.

**known statistical model (discrete)**	**analytical conclusions**				
	**power law**	**power-law tail**	**Poisson**	**geometric**	**none**
power law	0.90	0.97	0.00	0.00	0.10
Poisson	0.00	0.00	0.87	0.00	0.13
geometric	0.01	0.29	0.37	0.74	0.01

Probabilities of identifying data models from random samples drawn from synthetic data with known parameter values based on values derived from the empirical data. For example, reading row one, from left to right, the table says that given synthetic power-law distributions with known parameter values comparable to those derived from the empirical data, there is a 90% chance that the procedure used here would correctly identify them as power-law distributions, a 97% chance that it would identify a power-law distribution in the upper tail, a 0% chance that it would identify a Poisson or geometric distribution, and a 10% chance that it would spuriously fail to find a plausible fit to any of the statistical models under consideration.
